# Supplementary material for: Modified Polymer Surfaces: Thin Films of Silicate Composites via Polycaprolactone Melt Fusion
Source: Int J Mol Sci. 2022 Aug 15;23(16):9166. doi: 10.3390/ijms23169166 (PMC9409180; doi:10.3390/ijms23169166)
Supplement: Supplementary file 1 [file ijms-23-09166-s001.zip › ijms-1842754-supplementary.pdf]

Supplementary data.

Modified polymer surfaces: Thin film of silicate composite via polycaprolactone melt fusion

Eva Skoura[1], Peter Boháč[1,2], Martin Barlog[1,2], Helena Palková[1], Martin Danko[3], Juraj Šurka[4], Andreas Mautner[5], Juraj Bujdák[1,6]*

[1]Institute of Inorganic Chemistry, Slovak Academy of Sciences, Dúbravská cesta 9, Bratislava, SK- 845 36, Slovak Republic

[2]Centre of Excellence for Advanced Materials Application, Slovak Academy of Sciences, Bratislava, SK- 845 11, Slovak Republic

[3]Polymer Institute, Slovak Academy of Sciences, Bratislava, SK-845 41, Slovak Republic

[4] Earth Science Institute, Slovak Academy of Sciences, Ďumbierska 1, Banská Bystrica, SK-97411, Slovakia

[5] Polymer and Composite Engineering (PaCE) group, Department of Materials Chemistry, Faculty of Chemistry, University of Vienna, Währinger Str. 42, 1090 Wien, Austria

[6]Department of Physical and Theoretical Chemistry, Faculty of Natural Sciences, Comenius University in Bratislava, Bratislava, SK-842 15, Slovak Republic

* corresponding author

e-mail: juraj.bujdak@savba.sk

Section S1. Basic characterization of the polymer

The molar mass of PCL was estimated by gel permeation chromatography (GPC) using trifluoroethanol (TFE) as eluent with the addition of 0.1M potassium trifluoroacetate or tetrahydrofuran (THF). GPC system consists of Shimadzu LC-20 AT pump, Shimadzu refractive index detector and two PPS PFG 5 µm columns (*d* = 8 mm, *l* = 300 mm; 100 Å + 1000 Å) for TFE or three PSS SDV 5 µm columns (*d* = 8 mm, *l* = 300 mm; 100 Å + 1000 Å + 10000 Å) for THF at 25 °C. Poly(methyl methacrylate) and polystyrene standards (PSS, Germany) were used for calibration in TFE and in THF, respectively.

Thermal properties of neat PCL and composites were investigated by differential scanning calorimetry (DSC) using Discovery DSC (TA Instrument, Eschborn, Germany). 2.5-4.5 mg of samples were heated from −50 °C to 200 °C at a rate of 10 K min^{-1}, followed by cooling to −50°C. The samples were then heated again to 300°C at the same rate. The thermal stability was investigated by thermo-gravimetric analysis (TGA) using high resolution modulated TGA (Discovery TGA, TA Instruments, Eschborn, Germany). Approximately 2.66 mg of a sample was heated in an N_2 atmosphere from 30 to 700°C at a rate of 10 K min^{-1} and a gas flow rate of 25 mL min^{-1}.

The determination of PCL molar mass using GPC analyses provided the data shown in Table S1. Average number (M_n) and weight molar mass (M_w) determined in two solvents indicates a relatively large size of the polymer chains. The magnitude of M of ~10^5 is proportional to the mass of approximately ~1000 caprolactone monomeric units. The dispersity index was *Đ*<2.

Table S1a. Molar mass parameters for polycaprolactone characterization.

Solvent / standards	M_n / g mol^{-1}	M_w / g mol^{-1}	*Đ*
tetrahydrofurane / polystyrene	117400	171100	1.50
trifluoroethanol / polymethylmethacrylate	82500	161700	1.96

Thermal analysis measurements were carried out to characterize the polymer (see Supplementary data Fig. S1a and Fig. S1b). The thermal parameters of PCL such as melting temperature (T_m) and the changes of enthalpies of phase transitions are shown in Table S1b. Crystallization temperatures are not shown, since they were close to the room temperature and could not be accurately determined by these measurements. The composite sample PCL/M10 was also measured for comparison. An endothermic peak helped to identify the melting temperature at about 60°C for the neat PCL and the composite. The change of the enthalpy of polymer melting (ΔH_m) was about 78 J g^{-1}. The negative value indicating an exothermal reaction was obtained for an opposite process representing the crystallization of the polymer, but its absolute value was significantly reduced. Most probably the reverse transition was not completed by the cooling to the room temperature. Thermal stability of the composite was determined by

TGA analysis. The results showed significantly promoted decomposition of the PCL/MB10 composite. While neat PCL was stable almost up to temperature 350 °C, PCL/MB10 composite began to degrade rapidly above temperature 200 °C (Fig. S1b). It was observed that clay fillers can reduce oxygen diffusion into the polymer matrix and thus enhance its thermal stability [1]. On the other hand, residual metal ions present at the edges of saponite particles can act as transesterification catalyst, what contributes to polyester degradation [2]. The complex system PCL/MB10 containing Sap and MB organic dye with not completely mixed filler in the whole volume of polymer matrix exhibit catalytic affect of PCL degradation.

[1] P. J. Jandas, S. Mohanty, and S. K. Nayak, 'Morphology and Thermal Properties of Renewable Resource-Based Polymer Blend Nanocomposites Influenced by a Reactive Compatibilizer', ACS Sustainable Chem. Eng., 2, 3, pp. 377–386, 2014, doi: 10.1021/sc400395s

[2] M. Danko, M. Basko, S. Ďurkáčová, A. Duda, J. Mosnáček, Functional Polyesters with Pendant Double Bonds Prepared by CoordinationInsertion and Cationic Ring-Opening Copolymerizations of Caprolactone with Renewable Tulipalin A, *Macromolecules,* **2018**, 51, 3582-3596. Doi: 10.1021/acs.macromol.8b00456.

Table S1b. The thermal parameters obtained by the differential scanning calorimetry and thermogravimetric measurements. The plots are available in Fig. S1a, S1b.

**Sample**	**Heating**		**Cooling**
	T_m (°C)	ΔH_m (J g^{-1})	ΔH_c (J g^{-1})
PCL	63	78	-57
PCL/MB10	61	77	-56

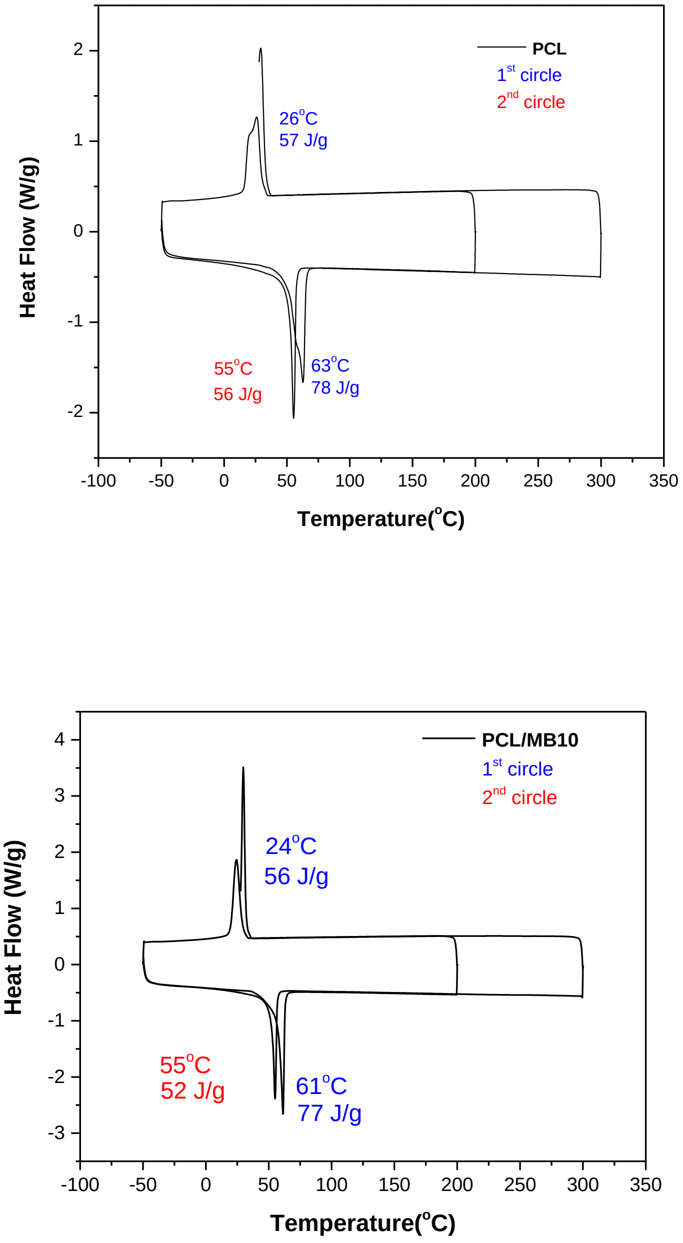

Figure S1a. Differential scanning calorimetry (DSC) of neat PCL (upper) and the composite PCL/MB10 (lower).

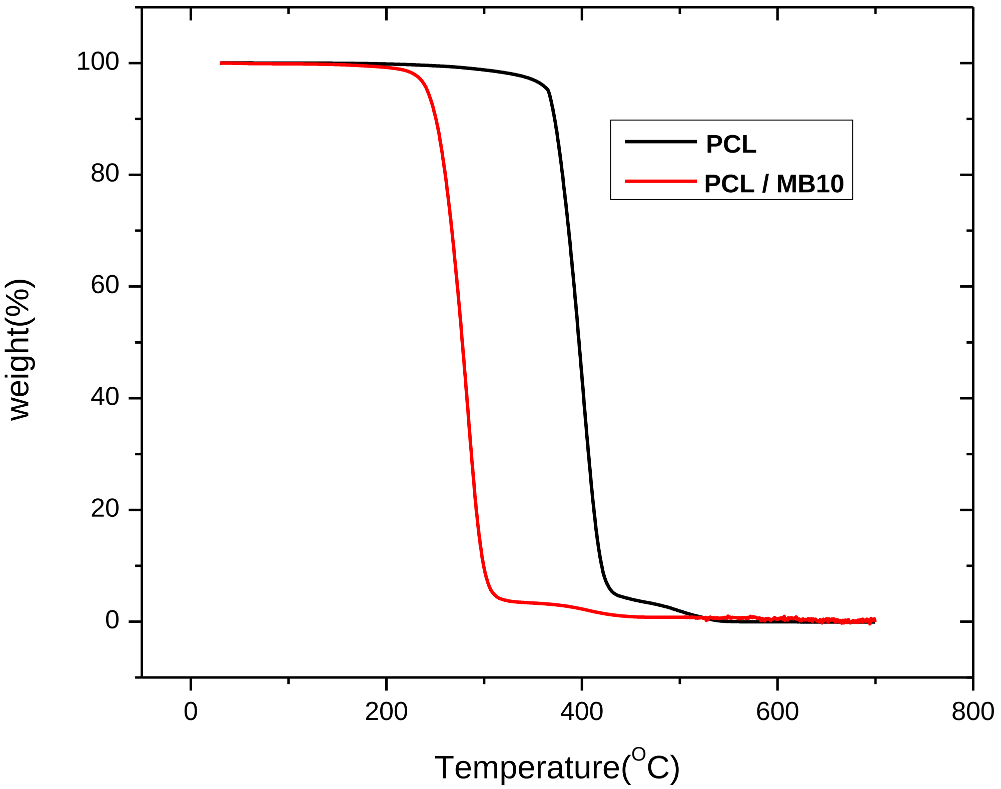

Figure S1b. Thermo-gravimetric analysis (TGA) of neat PCL and the composite PCL/MB10.

The results show the presence of MB10 catalyzes the decomposition of the polymer, which for the composite occurs at lower temperatures.

Section S2. Scanning electron microscopy images.

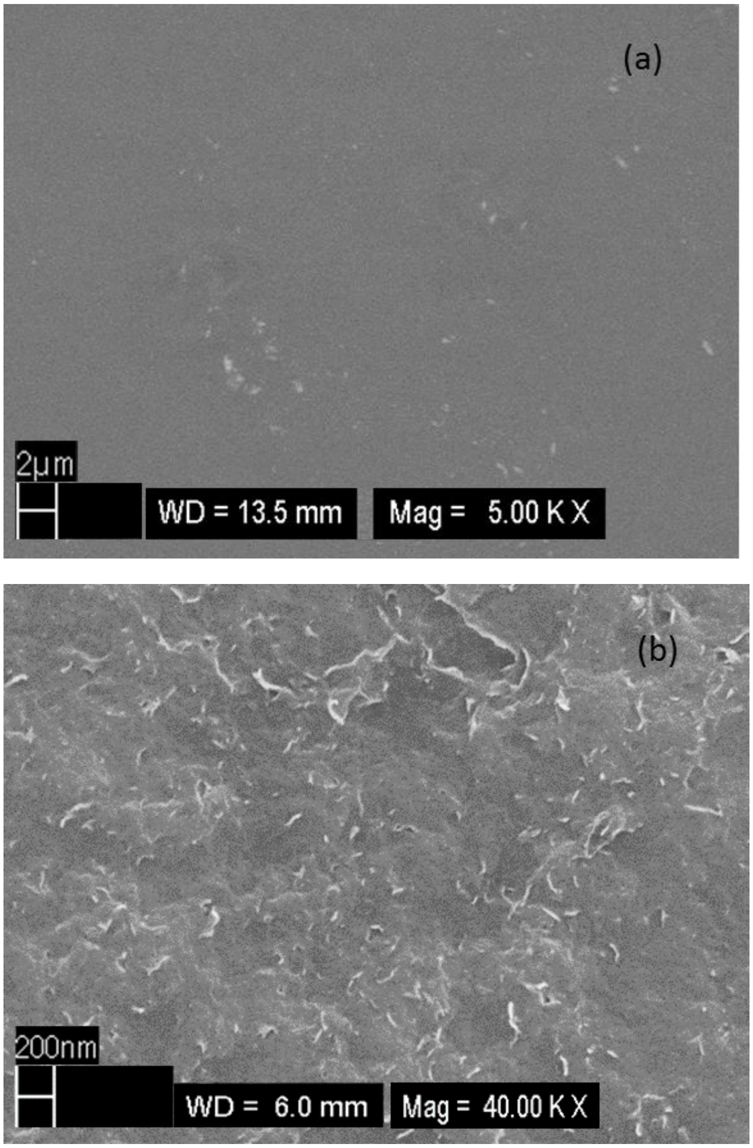

Figure S2. SEM images of surfaces of neat PCL (a) and the composite PCL/MB10 (b)

Section S3. Chemometric analysis of infrared spectra

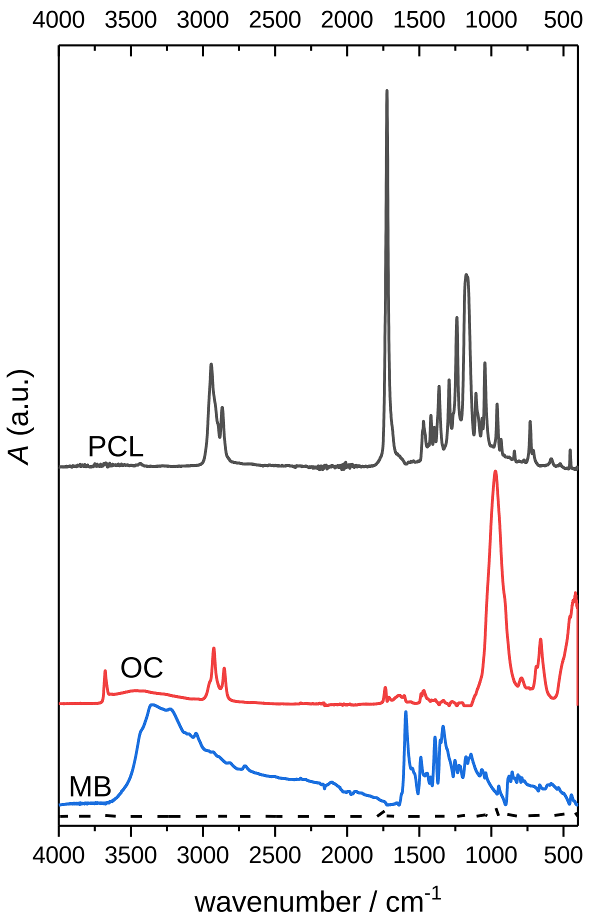

Figure S3. The spectral profiles of significant components calculated from the matrix of the infrared spectra using multivariate curve resolution - alternating least squares.

The calculated PCL_{MCR}, MB_{MCR}, and OC_{MCR} profiles are for simplicity assigned as PCL, OC, and MB respectively. The dashed line denotes the profile of variable residuals obtained by the calculation.

Section S4. X-ray photoelectron spectroscopy

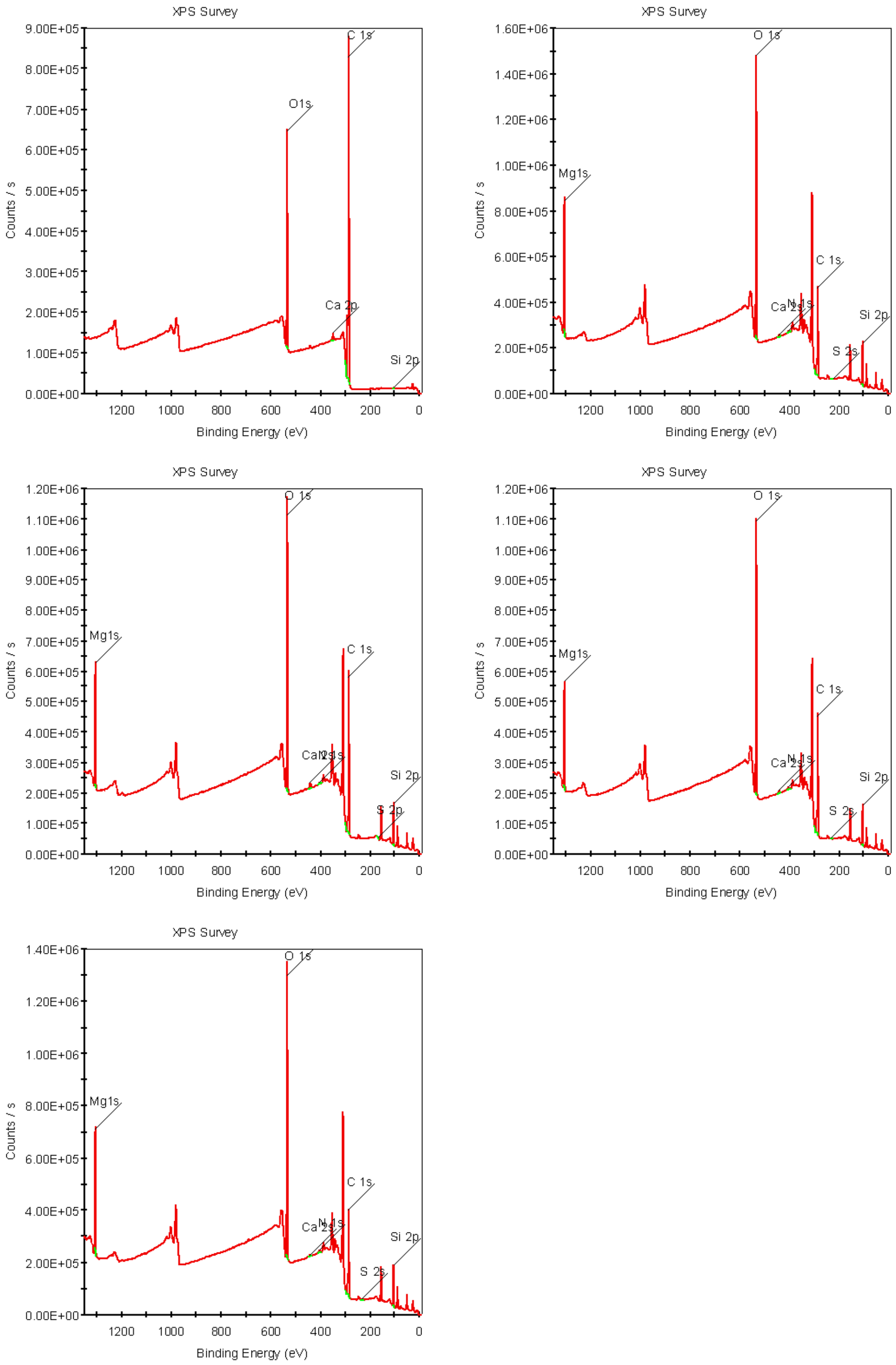

Figure S4 X-ray photoelectron spectra: survey spectra of PCL, PCL/OC, PCL/MB4, PCL/MB7, and PCL/MB10 (from left to right and top to bottom)

Table S2: Surface elemental composition [at-%] of PCL, PCL/OC, PCL/MB4, PCL/MB7, and PCL/MB10 from survey spectra.

Elelement	PCL	PCL/OC	PCL/MB4	PCL/MB7	PCL/MB10
C	77.94%	30.92%	41.74%	39.28%	31.10%
O	21.37%	43.72%	37.41%	39.60%	43.50%
Si	0.29%	13.93%	11.18%	11.86%	13.87%
N	-	0.37%	0.24%	0.72%	0.96%
S	-	0.05%	0.01%	0.12%	0.13%
Ca	0.38%	0.51%	1.31%	0.70%	0.58%
Mg	-	10.49%	8.10%	7.72%	9.86%

Section S5. Optical properties of the colloidal precursors.

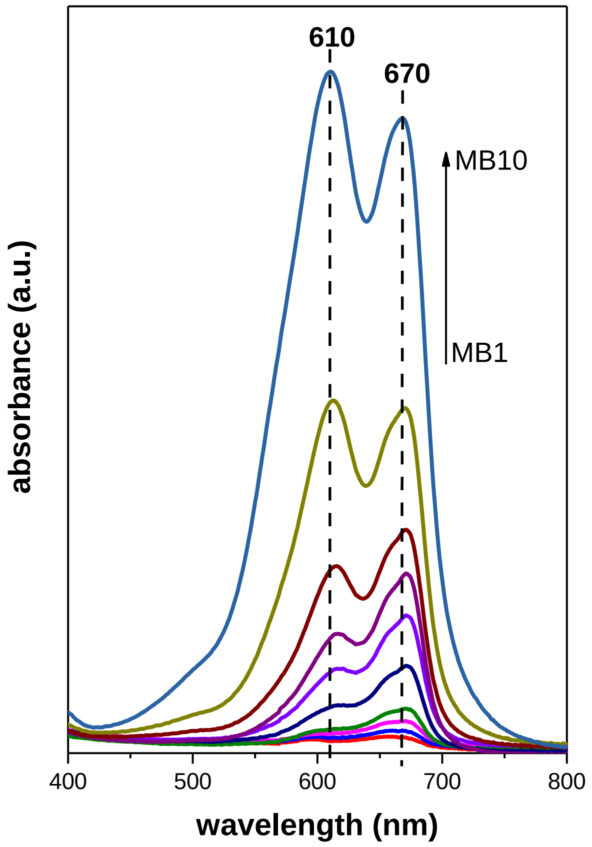

Figure S5. UV-Vis spectra of organoclay colloidal dispersions used as precursors of methylene blue/organoclay films.

Section S6. Methylene blue concentration in the films

Table S3: Average surface and volume concentrations of methylene blue in thin films of functionalized organoclays and composites on PCL membranes.

Samples									
MB1	MB2	MB3	MB4	MB5	MB6	MB7	MB8	MB9	MB10
Surface concentration / mol cm^{-2}									
2×10^{-9}	4×10^{-9}	5×10^{-9}	10^{-8}	2×10^{-8}	4×10^{-8}	5×10^{-8}	10^{-7}	2×10^{-7}	4×10^{-7}
Volume concentration / mol L^{-1}									
0.004	0.008	0.01	0.02	0.04	0.08	0.1	0.2	0.4	0.8

The parameters were calculated considering the area and thickness of the films of 2 cm^2 and 5 μm, respectively.
